# Magnetic resonance parkinsonism indices and interpeduncular angle in idiopathic normal pressure hydrocephalus and progressive supranuclear palsy

**DOI:** 10.1007/s00234-020-02500-1

**Published:** 2020-07-24

**Authors:** Lorenzo Ugga, Renato Cuocolo, Sirio Cocozza, Giuseppe Pontillo, Andrea Elefante, Mario Quarantelli, Caterina Vicidomini, Maria Francesca De Pandis, Giovanna De Michele, Alessandra D’Amico, Oreste de Divitiis, Arturo Brunetti

**Affiliations:** 1 Department of Advanced Biomedical Sciences, University of Naples “Federico II”, Via Pansini, 5, 80131 Naples, Italy; 2 Institute of Biostructure and Bioimaging, National Research Council, Naples, Italy; 3 San Raffaele Rehabilitation Institute, Cassino, Italy; 4 Department of Neurosciences and Reproductive and Odontostomatological Sciences, University of Naples “Federico II”, Naples, Italy

**Keywords:** Normal pressure hydrocephalus, Progressive supranuclear palsy, Magnetic resonance imaging, Magnetic resonance parkinsonism index, Interpeduncular angle

## Abstract

**Purpose:**

The clinical presentation of idiopathic normal pressure hydrocephalus (iNPH) may overlap with progressive supranuclear palsy (PSP). The Magnetic Resonance Parkinsonism Index (MRPI), MRPI 2.0, and the interpeduncular angle (IPA) have been investigated to differentiate PSP from healthy controls (HC) and other parkinsonisms. We aimed to assess equivalences and differences in MRPI, MRPI 2.0, and IPA in iNPH, PSP, and HC groups.

**Methods:**

We retrospectively recruited 99 subjects (30 iNPH, 32 PSP, 37 HC) from two institutions. MRI exams, acquired on either 1.5 T or 3 T scanners, included 3D T1-weighted images to measure MRPI, MRPI 2.0, and IPA. Inter- and intra-rater reliability was investigated with the intra-class correlation coefficient (ICC), and the two one-sided *t* tests (TOST) procedure was used to assess these markers in iNPH, PSP, and HC.

**Results:**

For all the three measures, intra-rater and inter-rater ICC were excellent (range = 0.91–0.93).

In the comparison of iNPH and PSP with HC, differences for MRPI and MRPI 2.0 (*p* < 0.01 in all cases) and no equivalence (*p* = 1.00 in all cases) were found at TOST. iNPH and PSP MRPI showed no difference (*p* = 0.06) and no equivalence (*p* = 0.08). MRPI 2.0 was not equivalent (*p* = 0.06) and not different (*p* = 0.09) in the same two populations. PSP and HC IPA proved equivalent (*p* < 0.01) while iNPH IPA was different (*p* < 0.01) and not equivalent (*p* = 0.96 and 0.82) from both PSP and HC.

**Conclusion:**

MRPI and MRPI 2.0 significantly overlap in iNPH and PSP, with risk of misdiagnosis, and for this reason may not be helpful in the differential diagnosis.

**Electronic supplementary material:**

The online version of this article (10.1007/s00234-020-02500-1) contains supplementary material, which is available to authorized users.

## Introduction

Idiopathic normal pressure hydrocephalus (iNPH) is a potentially treatable syndrome characterized by a variable combination of impaired gait, cognition, and urinary dysfunction (urgency and incontinence) [1]. iNPH can be diagnosed by medical history, neurologic examination, and brain imaging with CT or MRI. An early diagnosis is essential to achieve an optimal treatment outcome and to avoid irreversible impairments. However, the differential diagnosis can be challenging, because the clinical spectrum of iNPH overlaps with that of other entities, especially atypical parkinsonisms. In particular, progressive supranuclear palsy (PSP) shares with iNPH some of the cardinal clinical features, i.e., gait dysfunction, postural instability with retropulsion, and cognitive impairment [2].

Clinically, PSP could be distinguished from iNPH based on other specific features, in particular the typical ocular motor dysfunction, characterized by supranuclear gaze palsy or slowing of vertical saccades. Nevertheless, the ocular motor dysfunction can be missing in the early stages of PSP, especially in non-Richardson’s phenotypes [2]. On the other side, the urinary dysfunction is a classical feature of iNPH, but can be a non-specific and frequent finding in a population older than 60 years [3].

To improve diagnostic accuracy of PSP and iNPH, various neuroimaging indices have been proposed in the last few years. Magnetic Resonance Parkinsonism Index (MRPI) has been introduced by Quattrone et al. in 2008 [4] to recognize patients with PSP, and has therefore proved useful in helping clinicians to consolidate the diagnosis based on clinical features. As an extension of this metric, the MRPI 2.0, including the measurement of the third ventricle width and of the frontal horn distance, has been more recently introduced, which showed a superior accuracy, as compared with MRPI, in differentiating PSP patients from those with early stage Parkinson’s disease (PD) [5]. Additionally, interpeduncular angle (IPA) has been proposed to differentiate PSP patients from other parkinsonisms, with discordant results [6, 7]. On the other hand, the callosal angle and Evans index have proven effective in helping the radiologist differentiate patients with iNPH [8].

These indices proved to be useful in distinguishing PSP and iNPH from healthy controls and from other neurodegenerative diseases, but less useful to distinguish PSP and iNPH between each other [9].

Given the clinical but also radiological similarities between these two diseases, the aim of our study was to evaluate different MRI measurements (MRPI, MRPI 2.0, IPA) between PSP, iNPH, and healthy controls (HCs).

## Material and methods

### Participants

The present work has been carried out in accordance with The Code of Ethics of the World Medical Association (Declaration of Helsinki) for experiments involving humans.

We retrospectively analyzed the digital records at two different institutions to find iNPH and PSP patients who underwent MRI exams between January 2014 and December 2018. In all cases, only retrospective, anonymized information was used for the study; therefore, individual written informed consent was waived by the local IRBs (Comitato etico Università Federico II, Naples, Italy; Comitato Etico Lazio 2, Rome, Italy). Their inclusion was based on a diagnosis of “probable” disease in accordance with international guidelines [2, 10–12], made by a movement disorder specialist. Exclusion criteria were unavailability of a 3D isotropic T1-weighted (T1w) sequence, artifacts on the images used for the analysis, or the presence of significant neurological comorbidities. We exclusively selected the first MRI exam undergone by each patient. In this manner, we assessed the usefulness of the MRPI indices and IPA at the time of initial diagnosis, the ideal clinical application of these biomarkers. Then, a group of HC previously enrolled in other studies at the same institutions and whose exams also included 3D isotropic T1w images, was selected for the analysis.

### MR data acquisition and analysis

MR examinations were performed on three different scanners (1.5 Tesla Gyroscan Intera, Philips, Eindhoven, The Netherlands; Magnetom Espree, Siemens Healthineers, Erlangen, Germany; 3 Tesla Magnetom Trio, Siemens Healthineers, Erlangen, Germany). A complete list of all acquisition details and parameters is available in the supplementary materials.

Using 3D T1w isotropic images, MRPI and MRPI 2.0 were calculated as previously described [4, 5]. In particular, the midbrain and pons areas, divided by a line passing through the superior pontine notch and the inferior edge of the quadrigeminal plate, were measured on midsagittal T1w MR images. Middle cerebellar peduncles (MCP) were identified on parasagittal views, while superior cerebellar peduncles (SCP) were measured on oblique coronal MR image tangent to the floor of the fourth ventricle. The 3rd ventricle width was measured on an axial slice generated at the level of both the anterior and posterior commissures by averaging three different measurements of the maximum linear distance between the lateral borders. The frontal horn distance was evaluated on the axial view showing their maximal dilatation, and the largest left-to-right width was measured. MRPI was calculated by multiplying the midsagittal area of the pons/midsagittal area of the midbrain ratio by the MCP width/SCP width ratio. MRPI 2.0 values were obtained by multiplying the MRPI value by the 3rd ventricle width/frontal horn width ratio.

**Fig. 1:**
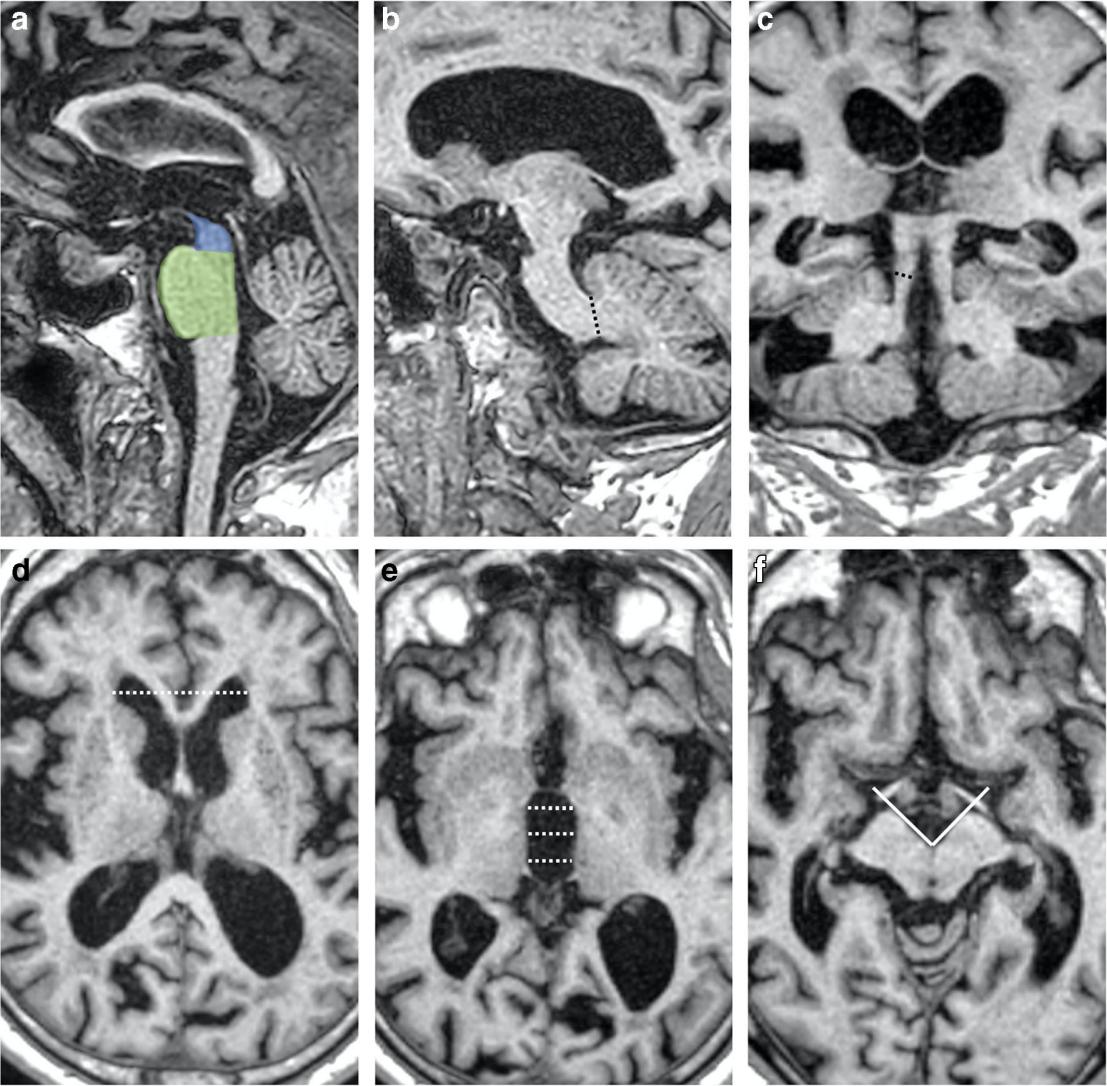
Sagittal (**a**–**c**) and axial (**d**–**e**) T1-weighted volumetric MR images of an iNPH patient showing sections on which MRPI and MRPI 2.0 measurements were performed. Midbrain and pons areas (**a**), middle (**b**), and superior (**c**) cerebellar peduncles thickness, frontal horn distance (d), and 3rd ventricle width (**e**) are depicted. Axial T1-weighted section on which interpeduncular angle was measured (**f**) is also shown

**Fig. 2:**
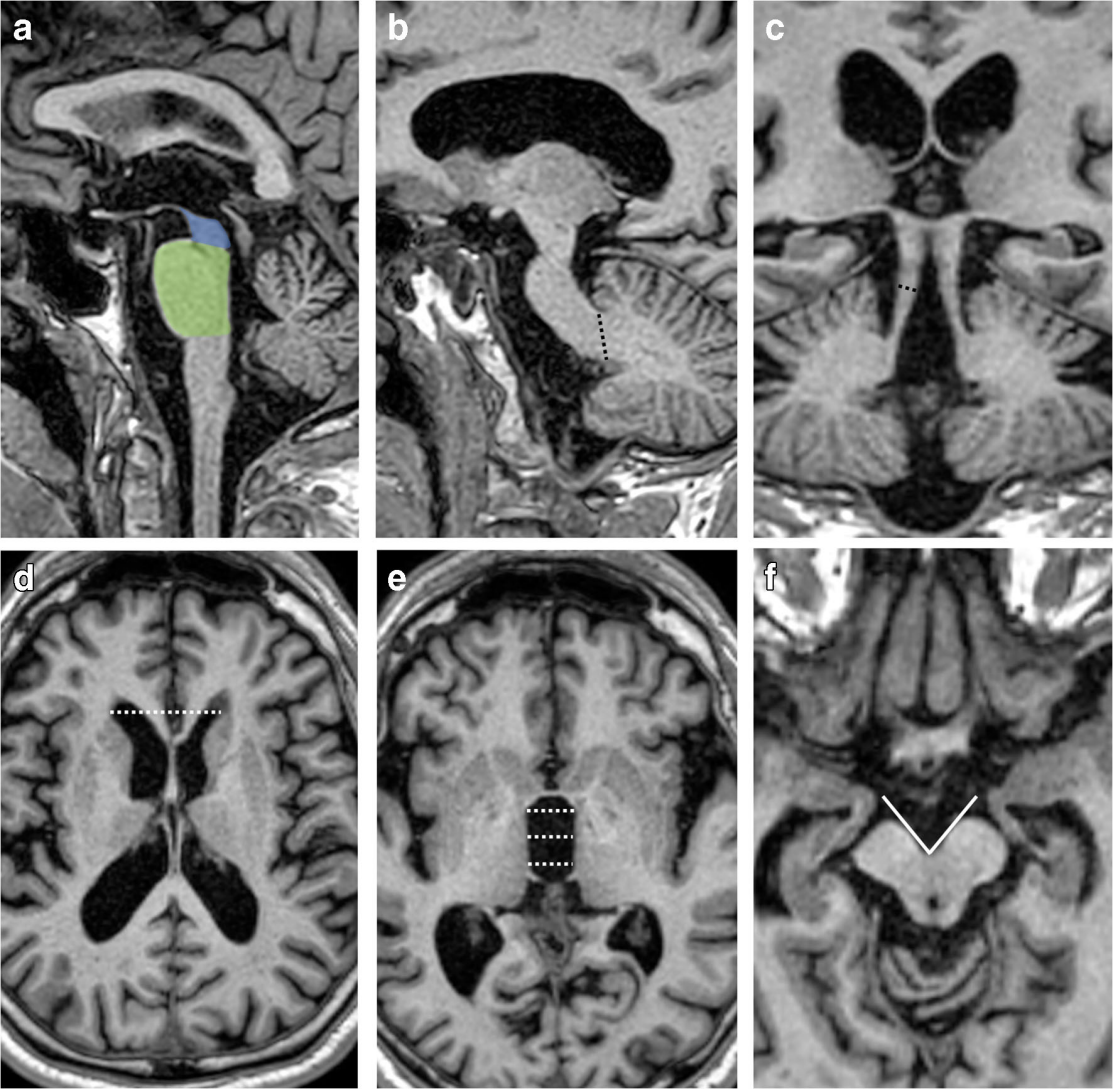
Sagittal (**a**–**c**) and axial (**d**–**f**) T1-weighted volumetric MR images of a PSP patient

Finally, the IPA was also calculated for all subjects, defined as the angle formed by the posterior half of the cerebral peduncles at the level of the mammillary bodies or immediately below [13]. Two examples of the obtained measures are available in Figs. 1 and 2 for an iNPH and a PSP patient, respectively.

Image analysis was performed independently by two raters (both with 8 years of experience).

### Statistical analysis

The obtained values were analyzed via equivalence testing following the two one-sided *t* tests (TOST) procedure corrected for multiple comparisons [14, 15]. This test takes into consideration the difference between “equivalent” and “not different.” The first implies confidence in stating there is no practical difference between the groups being compared, while the second that there was not sufficient evidence to determine they were different [16]. In TOST, the first one-sided test compares the mean with the lower equivalence bound and the second with the upper one, employing the larger *p* value to determine the result’s significance [14].

As populations were of different sample sizes, variance was not assumed as equal and Welch’s *t* test was employed. Equivalence bounds to use in the TOST were obtained through a preliminary power analysis with a desired power of 80%, an alpha value of 0.025, the sample size of the smaller group available in each comparison, and a pooled standard deviation obtained with Cohen’s formula. Differences between gender distribution were assessed by Fisher’s exact test, while continuous variables were compared using unpaired *t* tests.

The intra- and inter-rater reproducibility was calculated using the intra-class correlation coefficient (ICC). To assess the intra-rater reliability, one of the two raters performed a second evaluation after a 4-week washout period.

In detail, inter-rater reproducibility was calculated using a single rater, absolute agreement, two-way random effects model while the intra-rater with a single rater, consistency, two-way mixed effects one. The results were interpreted following the scale suggested by Koo and Li: poor (< 0.5), moderate (0.5–0.75), good (0.75–0.9), and excellent (> 0.9) [17].

All analyses were conducted using the R statistical software (R for Unix/Linux, version 3.4.4, the R Foundation for Statistical Computing, 2014) [18]; a *p* value < 0.05 was considered statistically significant with corrections for multiple comparisons when necessary.

## Results

Demographic data and MRI indices for the three groups are reported in Table 1. Mean age was 71.17 years (± 7.52) for iNPH, 72.19 (± 5.67) for PSP, and 69.09 (± 4.66) for control populations. Age distribution was normal for all groups (*p* = 0.35–0.50) without significant differences at ANOVA (*p* = 0.08). Similarly, no significant differences were found in terms of gender distribution (*p* = 0.10).

**Table 1:** Descriptive statistics of the clinical data and MRI indices for the population groups

	iNPH	PSP	Controls
Number	30	32	37
Age	71.17 (± 7.52)	72.19 (± 5.67)	69.09 (± 4.66)
Gender (M/F)	22/8	15/17	23/14
MRPI	15.23 (± 3.23)	17.01 (± 4.08)	8.63 (± 1.38)
MRPI 2.0	4.57 (± 1.37)	3.99 (± 1.30)	1.40 (± 0.48)
IPA (°)	83.50 (± 6.76)	75.38 (± 5.72)	75.53 (± 8.07)

Continuous variables are expressed as mean (± standard deviation)

Both intra-rater and inter-rater agreements proved to be excellent (ICC = 0.93 for MRPI, 0.92 for MRPI 2.0 and 0.92 for IPA; ICC = 0.92 for MRPI; 0.91 for MRPI 2.0 and 0.92 for IPA, respectively). The data for ICC analysis is available in the supplementary materials together with Bland-Altman plots for MRPI, MRPI 2.0, and IPA intra- and inter-rater agreement.

**Fig. 3:**
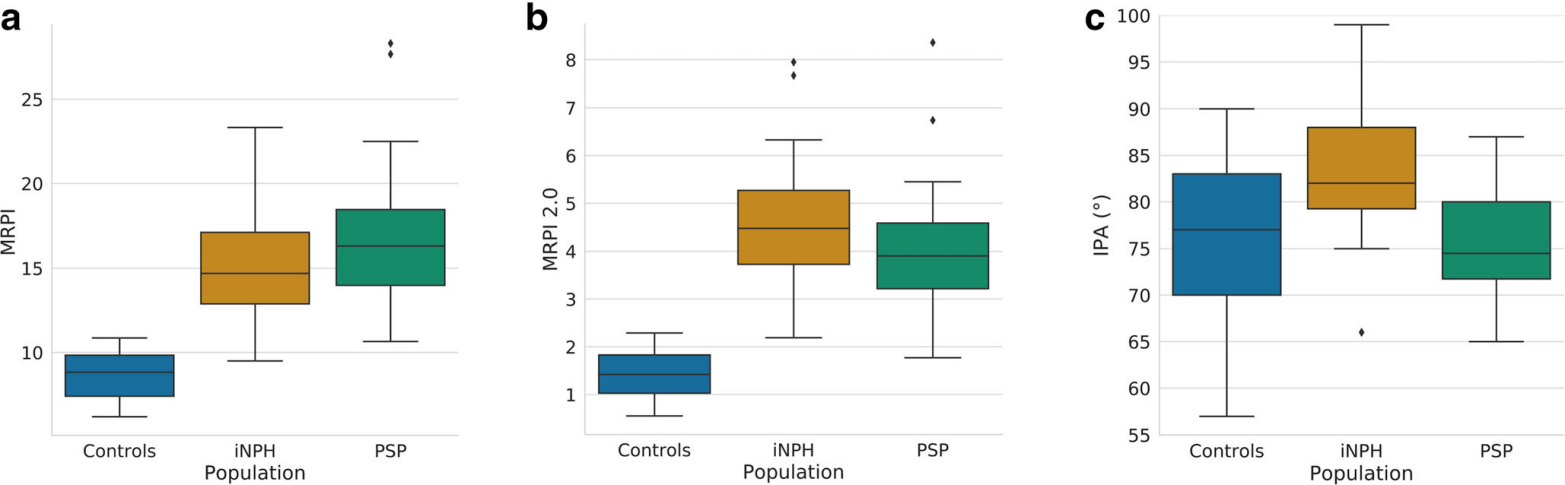
Box plot showing MRPI (**a**), MRPI 2.0 (**b**), and IPA (**c**) values distribution in healthy controls, iNPH, and PSP populations

Figure 3 shows the distribution of MRPI, MRPI 2.0, and IPA. In detail, iNPH patients had an average MRPI of 15.23 (± 3.23), MRPI 2.0 of 4.57 (± 1.37), and 83.50° IPA (± 6.76°). For the PSP group, these were respectively 17.01 (± 4.08), 3.99 (± 1.30), and 75.38° (± 5.72°). Finally, controls had 8.63 (± 1.38), 1.40 (± 0.48), and 75.53° (± 8.07°) averages for MRPI, MRPI 2.0, and IPA.

**Fig. 4:**
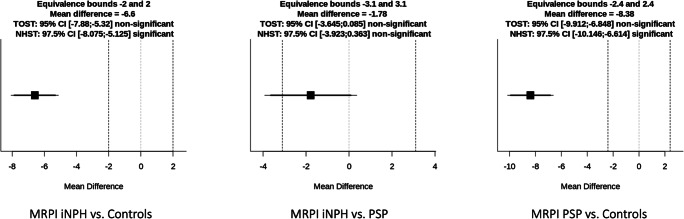
Mean difference plot depicting the equivalence testing results for MRPI

**Fig. 5:**
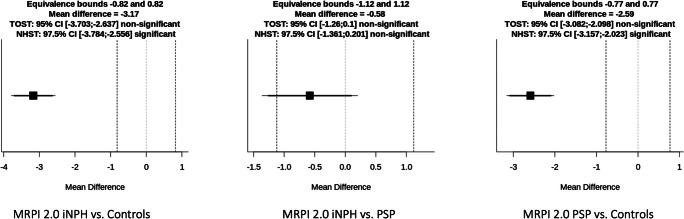
Mean difference plot depicting the equivalence testing results for MRPI 2.0

**Fig. 6:**
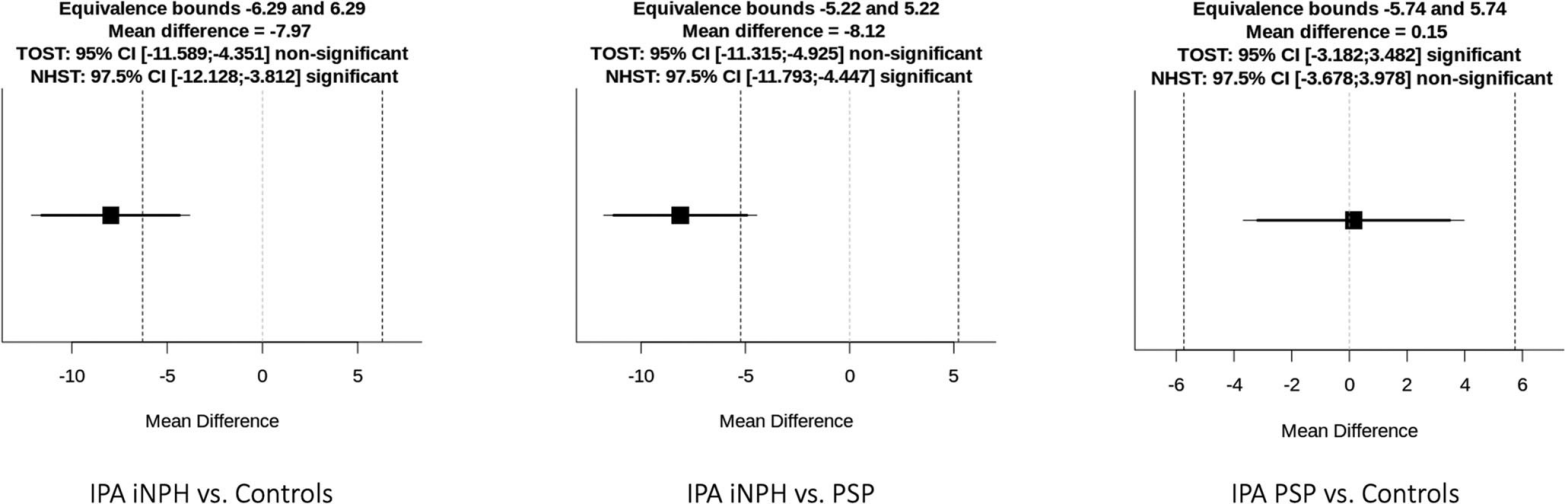
Mean difference plot depicting the equivalence testing results for IPA

Results of the comparisons performed with the corrected TOST procedure are illustrated in Table 2 and Figs. 4, 5, and 6. Briefly, no significant differences (*p* = 0.06) and no significant equivalence (*p* = 0.08) were found in MRPI score between iNPH and PSP patients. Similarly, MRPI 2.0 was non-equivalent (*p* = 0.06) and not different (*p* = 0.09) between these two patient groups. On the other hand, the comparison between iNPH patients and HC, as well as between PSP patients and HC, showed significant differences both for MRPI and MRPI 2.0 (*p* < 0.01 in all cases) and no equivalence (*p* = 1.00 in all cases). When the IPA measurements were evaluated, this metric proved to be equivalent between PSP patients and HC (*p* < 0.01), and not different (*p* = 0.93), while it was significantly higher in iNPH patients compared with both PSP and control groups (*p* < 0.01 in both cases), being not equivalent (*p* = 0.96 and 0.82, respectively).

**Table 2:** Results of the comparisons performed with the TOST procedure. Asterisks highlight statistically significant differences

Index	Groups	Equivalence test *p* value (lower and upper equivalence bounds)	Null hypothesis test *p* value
MRPI	iNPH vs PSP	0.08 (− 3.10; 3.10)	0.06
	iNPH vs controls	1.00 (− 2.00; 2.00)	1.2 × 10^−12^*
	PSP vs controls	1.00 (− 2.40; 2.40)	2.5 × 10^−13^*
MRPI 2.0	iNPH vs PSP	0.06 (− 1.12; 1.12)	0.09
	iNPH vs controls	1.00 (− 0.82; 0.82)	5.3 × 10^−14^*
	PSP vs controls	1.00 (− 0.77; 0.77)	5.1 × 10^−13^*
IPA	iNPH vs PSP	0.96 (− 5.22; 5.22)	4.2 × 10^−6^*
	iNPH vs controls	0.82 (− 6.29; 6.29)	4.1 × 10^−5^*
	PSP vs controls	6.7 × 10^−4^ (− 5.74; 5.74)*	0.93

## Discussion

In this study we evaluated the possible differences between iNPH, PSP, and HC in terms of different MRI metrics used in clinical practice. We found that both MRPI and MRPI 2.0 scores were not significantly different between iNPH and PSP patients, thus allowing us to suggest that these metrics could lead to a wrong neuroradiological evaluation in clinical practice.

iNPH represents the most common form of hydrocephalus in adults with a probable iNPH estimated prevalence of 0.2% in patients aged 70–79 years and 5.9% in patients aged 80 years and older, with no difference between men and women [19]. To date, the only effective treatment for iNPH is the shunt surgery [20, 21]. Nevertheless, only a part of iNPH patients achieve a significant clinical improvement after treatment, with different tests used to identify patients who are likely to respond to shunt surgery, including the tap test or CSF infusion testing [22]. It should be noted that from a clinical standpoint, different extra-pyramidal syndromes can overlap with findings found in iNPH. Among these, the most prominent differential diagnosis stands with PSP. Indeed, pure akinesia with gait freezing, accompanied by unsteadiness and falls, may be present in both groups of patients [23]. For this reason, the presence of normal pressure or obstructive hydrocephalus on imaging represents a mandatory exclusion criterion for a proper PSP diagnosis [2].

From a radiological standpoint, brain MRI changes in iNPH patients include ventriculomegaly, acute callosal angle, disproportionate changes in subarachnoid spaces with dilated Sylvian fissures, and narrow sulci and subarachnoid spaces at the vertex and medial/parafalcine region, defined as disproportionately enlarged subarachnoid-space hydrocephalus (DESH) [24, 25]. On the other hand, PSP is a progressive neurological disorder radiologically characterized by presence of midbrain atrophy and, to a lesser extent, supratentorial structures with ex vacuo dilation of the ventricle-cisternal system [26]. In detail, in a recent research Pyatigorskaya and colleagues performed a precise in vivo staging of neurodegeneration in PSP using quantitative multimodal MRI at 3 and 7 Tesla showing extensive volume decreases and diffusion changes in the midbrain, substantia nigra, subthalamic nucleus, globus pallidus, basal forebrain, locus coeruleus, pedunculopontine nucleus, and dentate nucleus, overlapping degrees of impairment in histological analyses [27].

In the last years, MRPI showed excellent performance in recognizing PSP patients, and in differentiating them from patients with PD, and for this reason, its clinical usage in auxiliary diagnosis of PSP is strongly recommended [28].

**Fig. 7:**
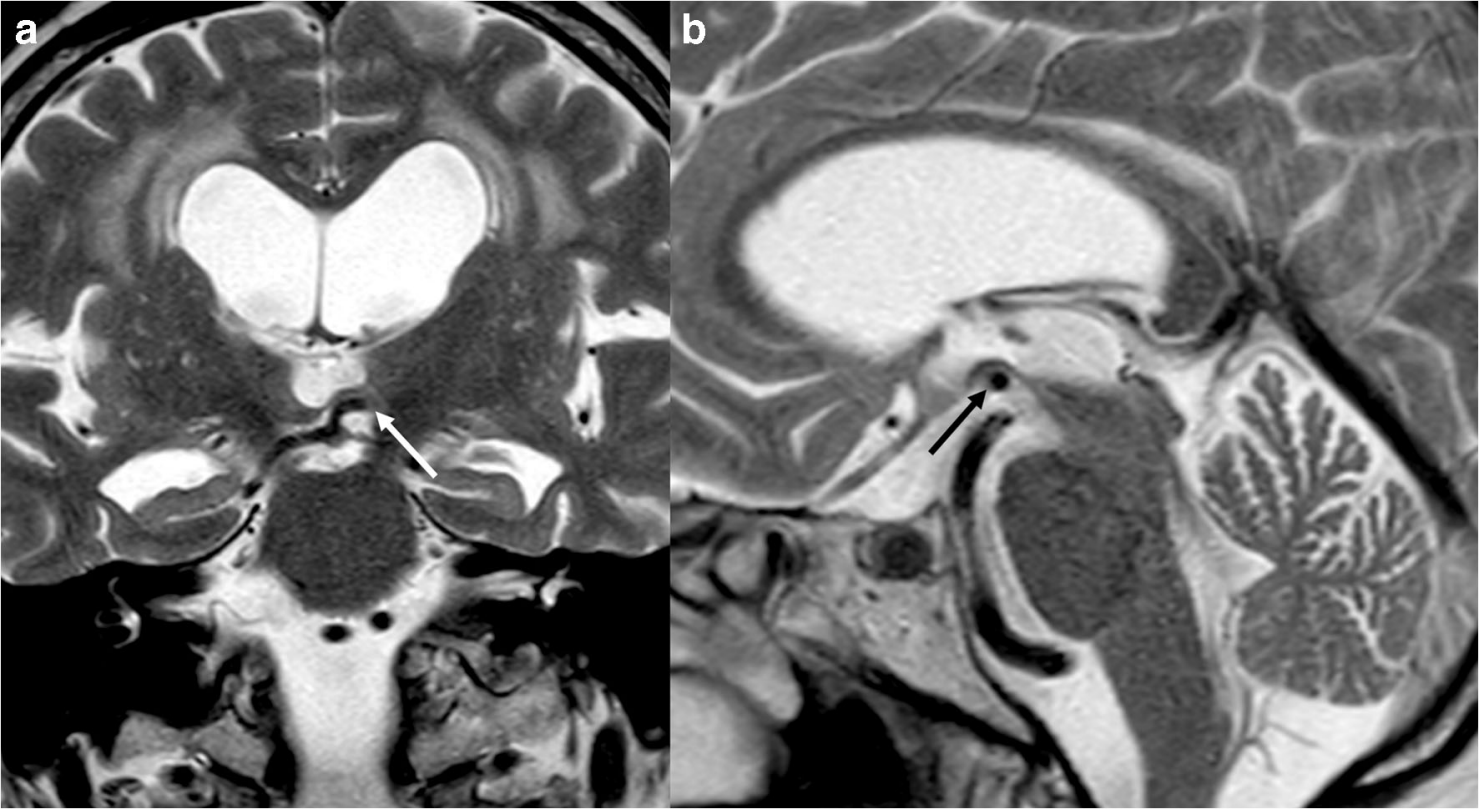
Coronal (**a**) and sagittal (**b**) T2-weighted images showing upper displacement of the third ventricle floor by the left posterior cerebral artery

Our results show no difference in MRPI between PSP and iNPH patients, leading to several considerations. Firstly, the increased size of the third ventricle in iNPH patients produces a widening of the cerebral peduncles, as demonstrated by the higher IPA values compared with those found in the HC. Given that the mesencephalic measurements are performed on the midsagittal slice, this may lead to an underestimation of the mesencephalic volume in iNPH patients. Even the inclusion of ventricular dilation markers in MRPI 2.0, compared with MRPI, does not solve this overlap in imaging findings, as shown in our results. Volume-based indices might be able to effectively quantify mesencephalic atrophy or superior cerebellar peduncle volume, even though their use in daily clinical practice is still limited [29]. Furthermore, the presence of a tortuous posterior circulation in some older iNPH patients leading to an upper displacement of the third ventricle floor by posterior cerebral arteries may contribute to alter the midsagittal mesencephalic morphology. This might produce flattening or concave outline to the superior aspect of the midbrain, which should be upwardly convex, possibly mimicking the hummingbird sign of PSP patients (Fig. 7).

In regard to the statistical power of our findings, it should be noted that the equivalence bounds were calculated as to ensure an 80% power. We wish to highlight that the resulting values were above the suggested cut-off proposed by Quattrone and colleagues both for MRPI and MRPI 2.0 in the differential diagnosis between PSP-P and HC (respectively 11.34 and 2.18) [5]. This supports the overlap in said scores between iNPH and PSP-P patients; both clearly increased compared with healthy subjects. In line with previous studies, we found an excellent reproducibility of all MRI metrics in our population [4, 5, 13], further corroborating the use of these measures in clinical practice.

A recent publication by Constantinides and colleagues investigated quantitative and qualitative MRI signs including MRPI in PSP, iNPH, and HC groups [30]. They report a difference in terms of MRPI between PSP and iNPH patients with a *p* value of 0.049. In our study, the same parameter showed no differences between these groups with a *p* value of 0.06. This could be explained by the different sizes of their iNPH group (*n* = 17 vs 30). Nonetheless, their conclusion further supports the imaging overlap between PSP and iNPH as none of the markers analyzed proved reliable in their differential diagnosis. In this setting, the differences we found in IPA between iNPH and both PSP and HC are of particular interest. This finding is further reinforced by the unequivocal equivalence of IPA values in the PSP and HC groups. For this reason, the IPA value might be a useful tool in the radiological evaluation of these patients, in addition to other already established measurements such as the callosal angle.

This study has some limitations which have to be pointed out. First of all, iNPH and PSP diagnoses were made by a movement disorder specialist with a “probable” level of diagnostic certainty, and not pathologically confirmed. This may have partially affected the results, since some patients with antemortem diagnosis of iNPH have been noted to have coexisting neurodegenerative pathologies including PSP on neuropathology [23]. Disease duration at the moment of MRI evaluation has not been taken into account. We are aware that disease duration and stage could impact the imaging presentation of these patients, while it has been reported that MRPI can detect abnormalities in very early stages of disease [31–33]; to address this issue the patient’s first MR study since clinical onset was considered. While the power analysis supports the validity of our findings, further studies on larger populations are obviously mandatory, to confirm our results. In particular, we think that a specific prospective investigation about the role of IPA to differentiate between iNPH and PSP patients is strongly warranted, given the findings of this study. Furthermore, the different Movement Disorder Society PSP subtypes were not considered in the present study, although it should be noted that in a recent study Picillo and colleagues showed that MRPI and MRPI 2.0 values are not significantly different among several PSP subtypes [34].

## Conclusion

Our study showed that MRPI and MRPI 2.0 scores may not be helpful in the differential diagnosis between PSP and iNPH, given the overlap of these metrics. On the other hand, IPA was generally higher in iNPH than in PSP patients and in HC; therefore, it demonstrated a useful additional marker to differentiate this potentially treatable condition.

## Electronic supplementary material

ESM 1 (DOCX 14 kb)

ESM 2 (XLSX 10 kb)

ESM 3 (XLSX 10 kb)

Supplementary Material Fig. 4 (PNG 369 kb)

Supplementary Material Fig. 5 (PNG 370 kb)

Supplementary Material Fig. 6 (PNG 357 kb)

Supplementary Material Fig. 7 (PNG 400 kb)

Supplementary Material Fig. 8 (PNG 370 kb)

Supplementary Material Fig. 9 (PNG 353 kb)

## Abbreviations

iNPH: Idiopathic normal pressure hydrocephalus
PSP: Progressive supranuclear palsy
MRPI: Magnetic Resonance Parkinsonism Index
PD: Parkinson disease
IPA: Interpeduncular angle
HC: Healthy controls
MCP: Middle cerebellar peduncles
SCP: Superior cerebellar peduncles
TOST: Two one-sided *t* tests
ICC: Intra-class correlation coefficient
