# Supplementary material for: Magnetic resonance parkinsonism indices and interpeduncular angle in idiopathic normal pressure hydrocephalus and progressive supranuclear palsy
Source: Neuroradiology. 2020 Jul 24;62(12):1657–65. doi: 10.1007/s00234-020-02500-1 (PMC7666671; doi:10.1007/s00234-020-02500-1)
Supplement: Supplementary file 1 — (DOCX 14 kb) [file 234_2020_2500_MOESM1_ESM.docx]

**Supplementary material**

**Supplemental Table 1**

Volumetric T1-weighted spoiled gradient echo acquisition parameters for different Institution scanners

|  | **Magnetic field** | **Sequence** | **TR/TE (ms)** | **TI (ms)** | **FOV (mm)** | **matrix** | **thk (mm)** | **flip angle** |
| --- | --- | --- | --- | --- | --- | --- | --- | --- |
| Gyroscan Intera, Philips, Eindhoven, The Netherlands | 1.5 Tesla | 3D TFE T1 | 7.60/3.5 | - | 256x256 | 256x256 | 1 | 8° |
| Magnetom Espree, Siemens Healthineers, Erlangen, Germany | 1.5 Tesla | MPRAGE | 1590/2.4 | 1000 | 260x260 | 192x192 | 1.3 | 8° |
| Magnetom Trio, Siemens Healthineers, Erlangen, Germany | 3 Tesla | MPRAGE | 1900/3.4 | 900 | 256x192 | 256x192 | 1 | 9° |

TFE: turbo field-echo; MPRAGE: Magnetization Prepared - RApid Gradient Echo; TR: repetition time; TE: echo time; TI: inversion time; FOV: field of view; thk: thickness.

**Supplementary figure 1**

Bland-Altman plots for MRPI intra-rater agreement.

**Supplementary figure 2**

Bland-Altman plots for MRPI 2.0 intra-rater agreement.

**Supplementary figure 3**

Bland-Altman plots for IPA intra-rater agreement.

**Supplementary figure 4**

Bland-Altman plots for MRPI inter-rater agreement.

**Supplementary figure 5**

Bland-Altman plots for MRPI 2.0 inter-rater agreement.

**Supplementary figure 6**

Bland-Altman plots for IPA inter-rater agreement.

**Intra-rater agreement data**

**Inter-rater agreement data**
