# Supplementary figures and images for: Magnetic resonance parkinsonism indices and interpeduncular angle in idiopathic normal pressure hydrocephalus and progressive supranuclear palsy
Source: Neuroradiology. 2020 Jul 24;62(12):1657–65. doi: 10.1007/s00234-020-02500-1 (PMC7666671; doi:10.1007/s00234-020-02500-1)

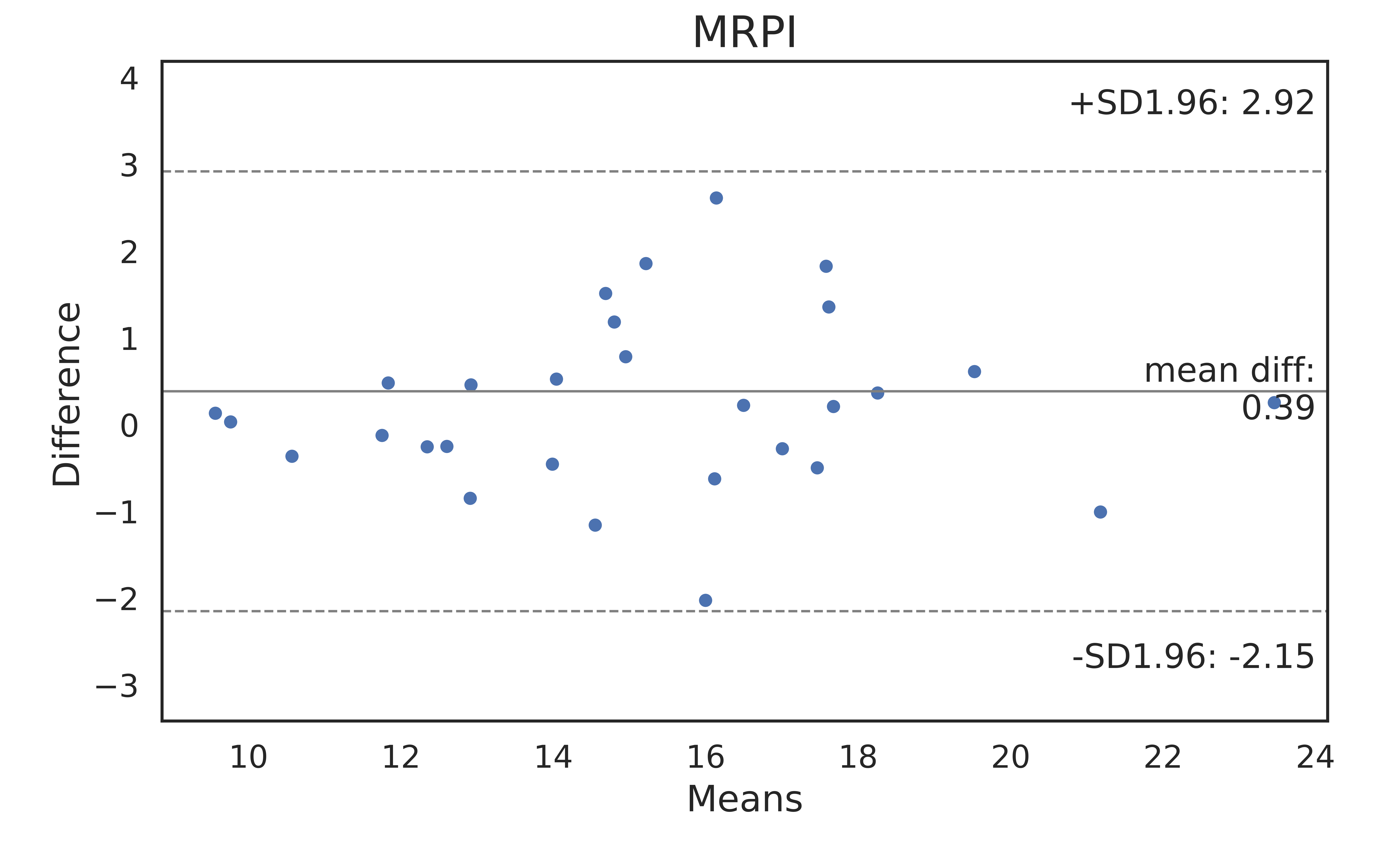

Supplement: Supplementary file 4 — Fig. 4 (PNG 369 kb) [file 234_2020_2500_MOESM4_ESM.png]

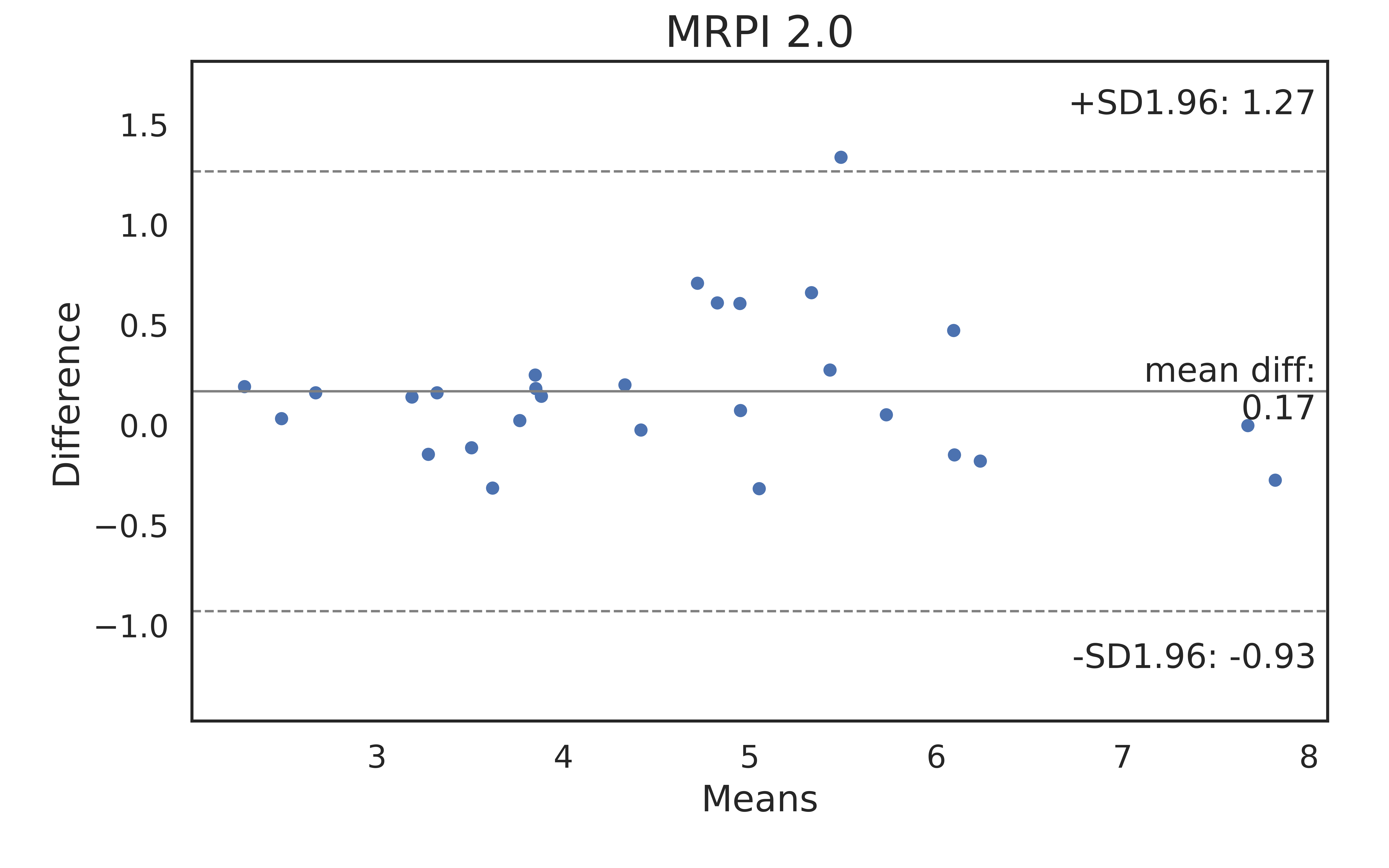

Supplement: Supplementary file 5 — Fig. 5 (PNG 370 kb) [file 234_2020_2500_MOESM5_ESM.png]

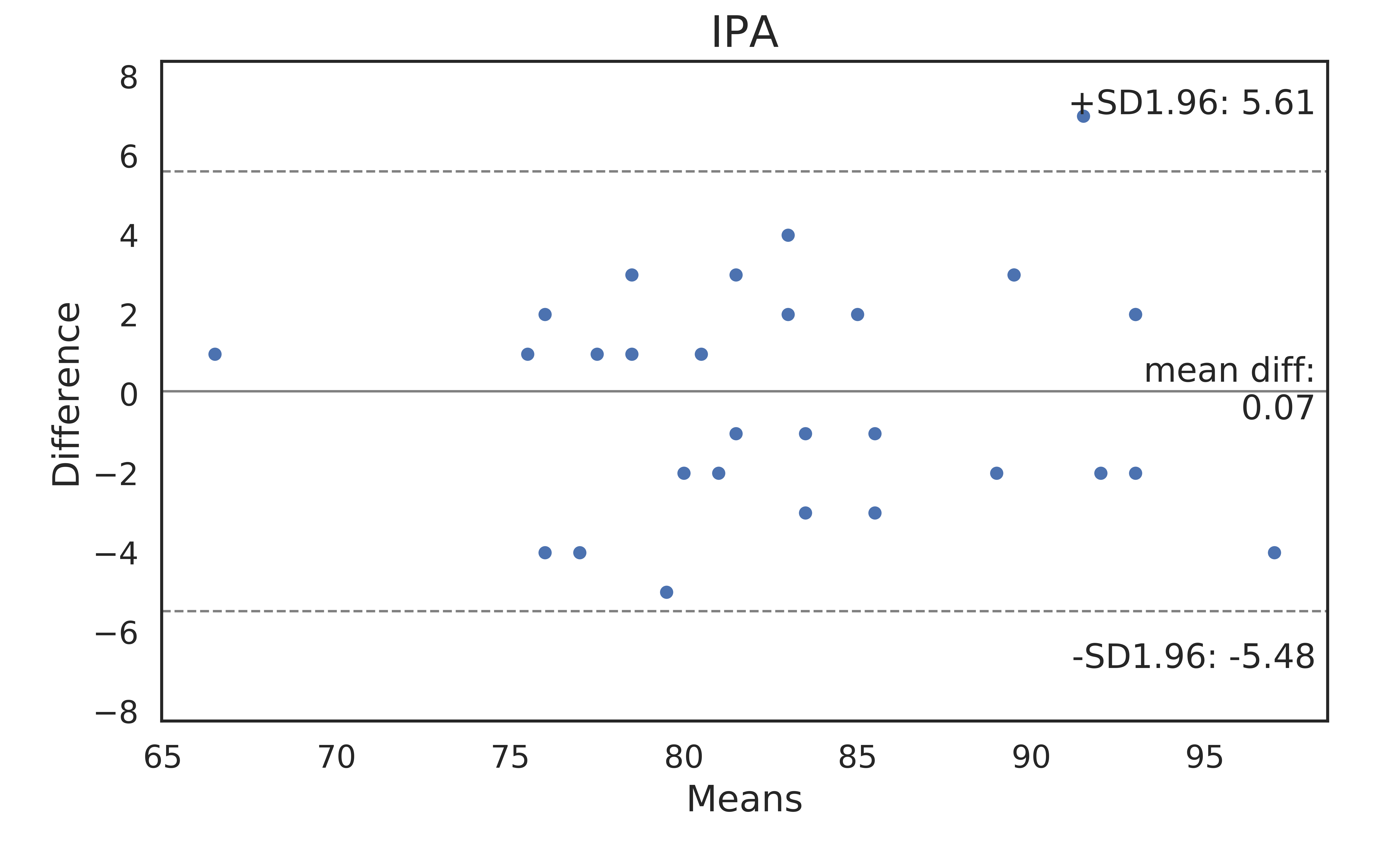

Supplement: Supplementary file 6 — Fig. 6 (PNG 357 kb) [file 234_2020_2500_MOESM6_ESM.png]

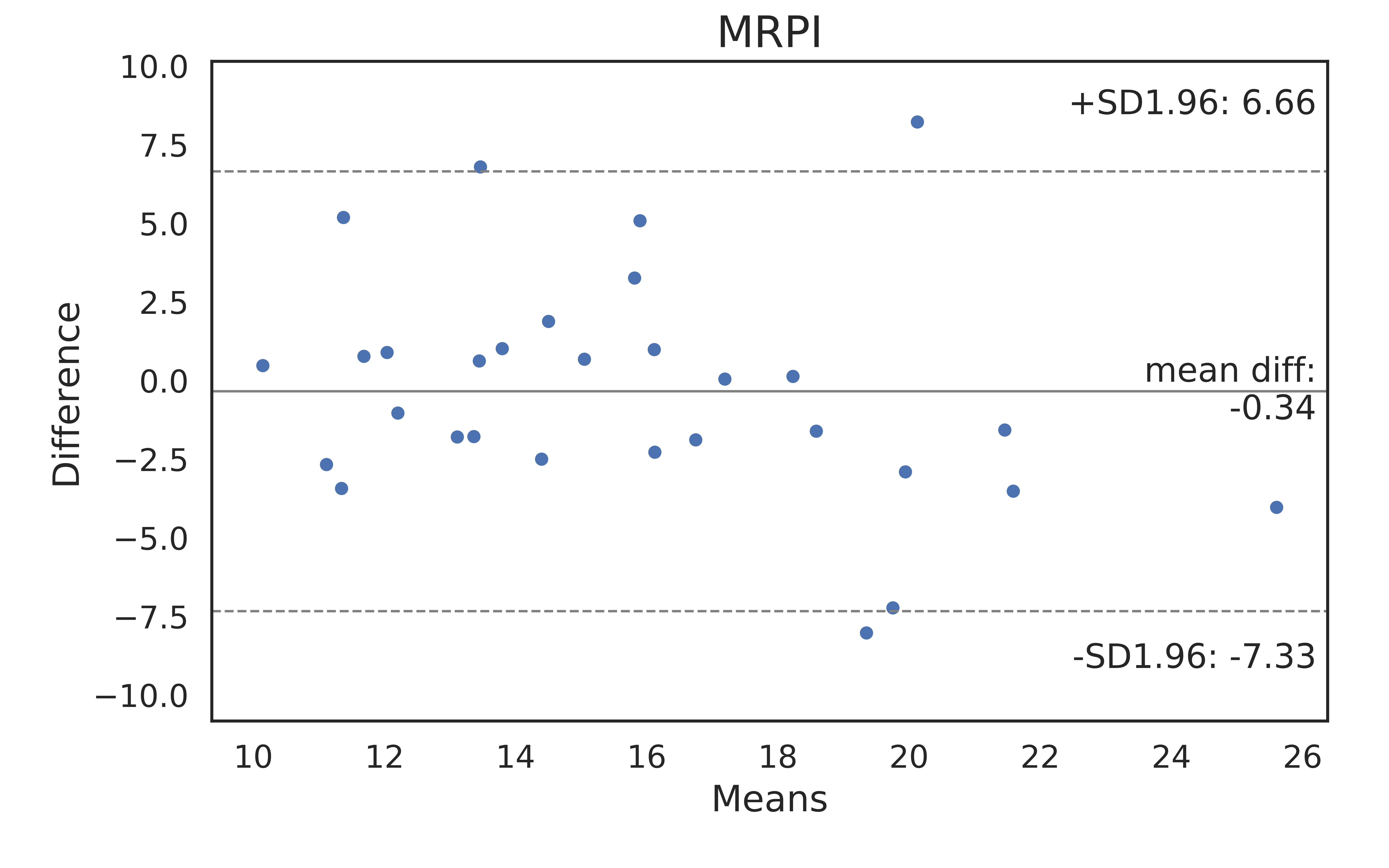

Supplement: Supplementary file 7 — Fig. 7 (PNG 400 kb) [file 234_2020_2500_MOESM7_ESM.png]

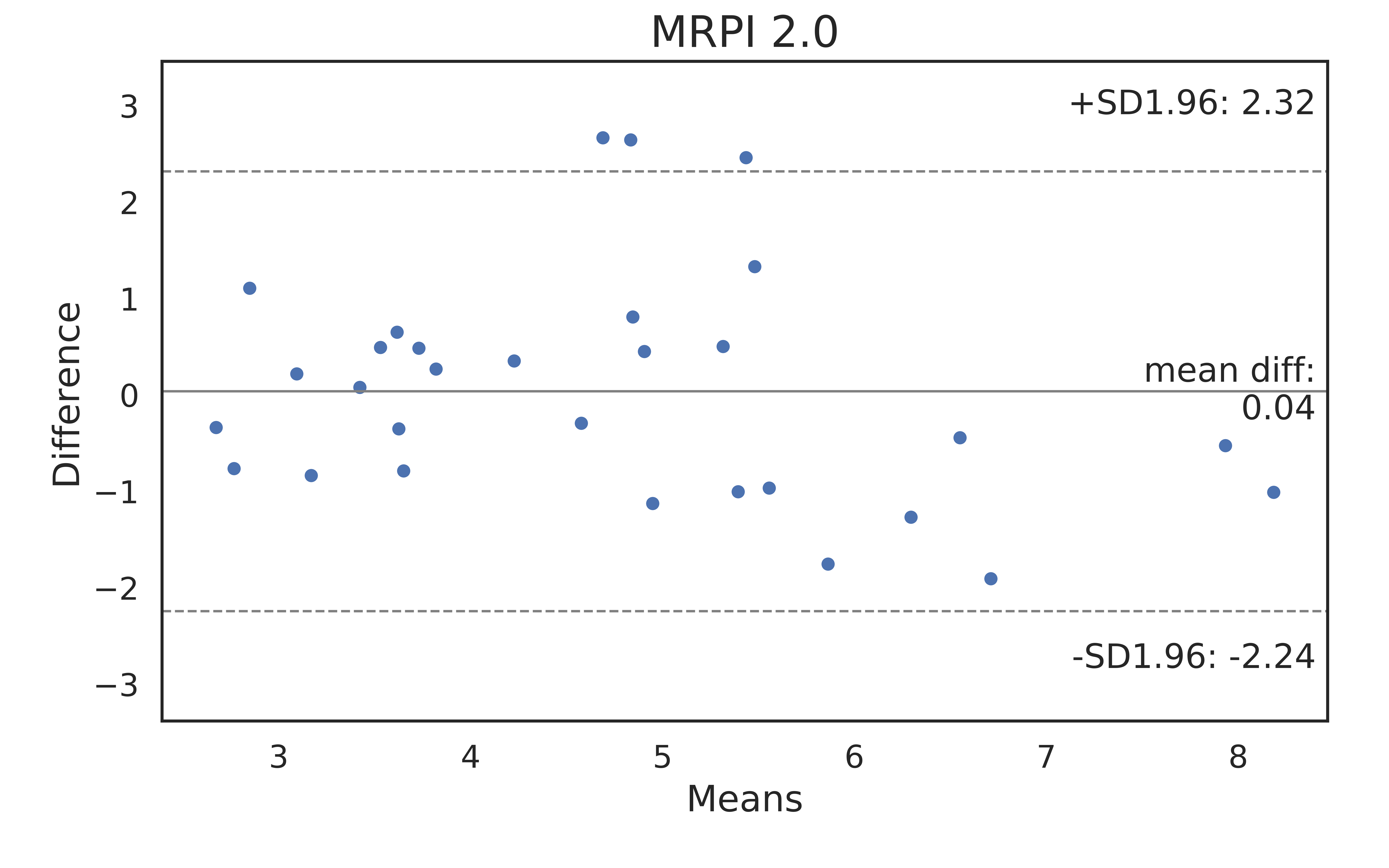

Supplement: Supplementary file 8 — Fig. 8 (PNG 370 kb) [file 234_2020_2500_MOESM8_ESM.png]

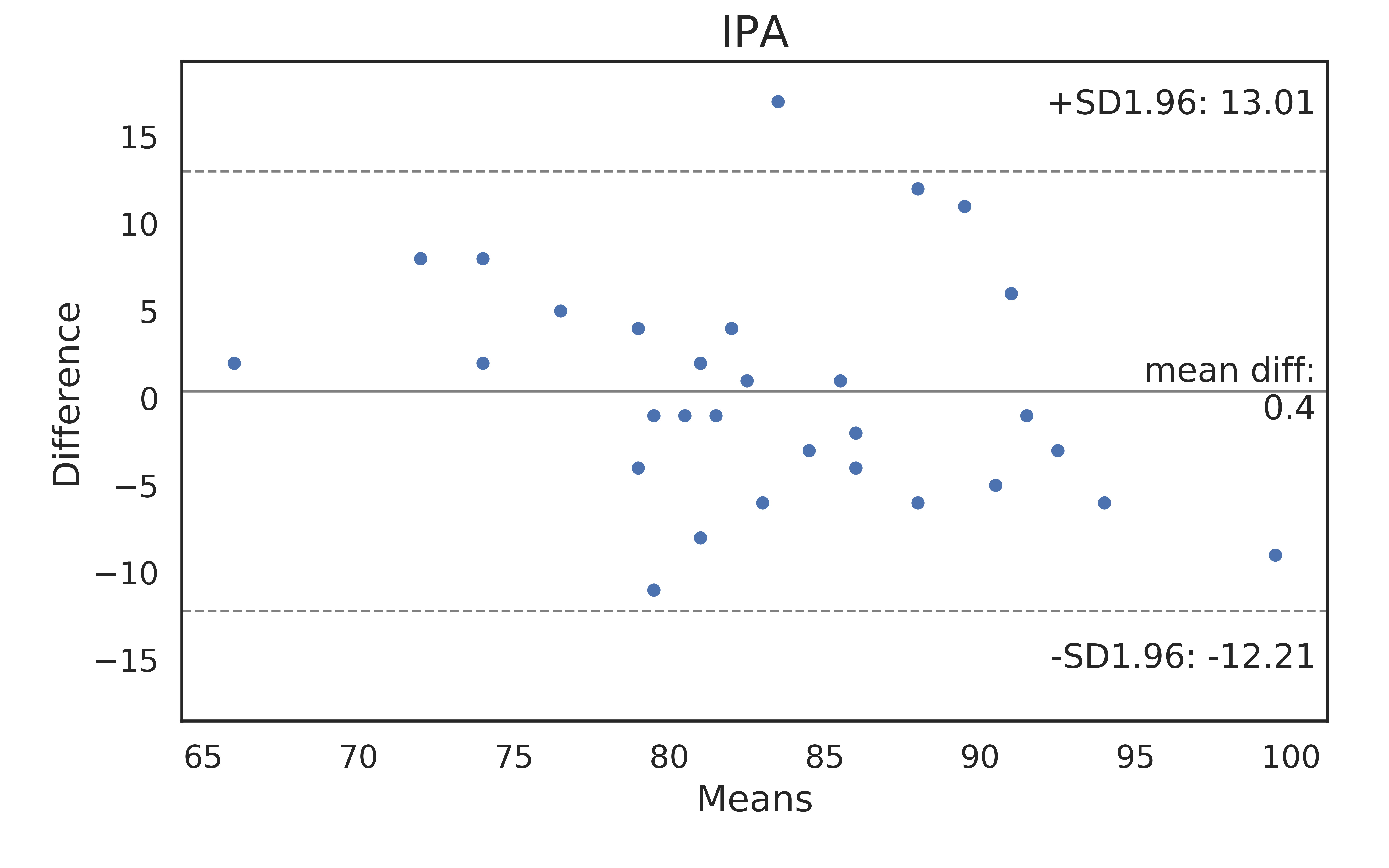

Supplement: Supplementary file 9 — Fig. 9 (PNG 353 kb) [file 234_2020_2500_MOESM9_ESM.png]
